# Chinese Propolis Inhibits the Proliferation of Human Gastric Cancer Cells by Inducing Apoptosis and Cell Cycle Arrest

**DOI:** 10.1155/2020/2743058

**Published:** 2020-07-22

**Authors:** Xia-sen Jiang, Hong-qing Xie, Chun-guang Li, Meng-meng You, Yu-fei Zheng, George Q. Li, Xiang Chen, Cui-ping Zhang, Fu-liang Hu

**Affiliations:** ^1^College of Animal Science, Zhejiang University, Hangzhou 310058, China; ^2^NICM Health Research Institute, Western Sydney University, Westmead, NSW 2145, Australia; ^3^Cai Jian Apiculture Company Limited, Huizhou 516000, China

## Abstract

Special Chinese propolis sourced from the Changbai Mountains (CBMP) in Northeast China is rich in specific flavonoids and phenolic acids and its bioactivity has not been reported. This study aimed to investigate the antiproliferative effect of CBMP on cancer cells and its molecular mechanisms. Different cancer cell lines were treated with the ethanol extracts of CBMP for 24 hours before the cell viability and mechanism measurements. The results showed CBMP had weak activities against human pancreatic cancer cell PANC1, human lung cancer cell A549, human colon cancer cell HCT116, human liver cancer cell HepG2, human bladder cancer cell T24, and human breast cancer cell MDA-MB-231, but it significantly inhibited the growth of human gastric cancer SGC-7901 cells, caused cell apoptosis and cell cycle arrest in S phase, with increased production of reactive oxygen species (ROS) and reduced mitochondrial membrane potential (MMP). The results indicate that Chinese propolis sourced from the Changbai Mountains selectively inhibits the proliferation of human gastric cancer SGC-7901 cells by inducing both death receptor-induced apoptosis and mitochondria-mediated apoptosis, and cell cycle arrest in S phase. These activities and mechanisms help understand the anticancer action of propolis and its active compounds.

## 1. Introduction

Propolis is a complicated resinous substance collected from different plants by honeybees (*Apis mellifera* L.) [1]. It has been widely used as a folk medicine since 3000 BC [2]. Over 300 compounds were identified in different types of propolis, and the chemical composition in propolis mainly depends on the plant sources [3]. Among different types of propolis, the poplar-type propolis is the most widely distributed one around the world, including Europe, North America, nontropical regions of Asia, North Africa, and Oceania [4–6]. The main biologically active compounds of poplar-type propolis are flavonoids and phenolic acids [7]. Propolis has a wide range of pharmacological activities, such as anti-inflammatory, antioxidant, and antimicrobial effects [8–12]. Moreover, the anticancer activity of propolis and its main compounds has been proved by both *in vitro* and *in vivo* experiments [10, 13–15].

Cancer is increasing prevalence worldwide and the second leading cause of human death [16]. Natural products have proven to be effective and safe in the treatment and prevention of cancers [17]. The anticancer property of propolis has been well demonstrated. For example, Chinese propolis and Brazilian propolis were shown to inhibit cell growth and increased apoptosis in human colon carcinoma HCT116 cells [18]. Propolis from Thailand and Turkey was also shown to induce DNA fragmentation and apoptosis or arrest the cell cycle of A549 cells and HeLa cells [19, 20]. In addition, the components of propolis, including prenylated flavanones, caffeic acid phenethyl ester (CAPE), and pinocembrin, were demonstrated to have different antitumor activities, such as chrysin-induced apoptosis and inhibition of cancer cell growth *in vitro* and *in vivo* [21–24].

The Changbai Mountains are one of the main mountain ranges in China, stretching throughout Northeast China, which has a wide variety of botanical resources [25], and this region is the main linden honey producing area in China. Our recent study showed that propolis sourced from the Changbai Mountains (CBMP) is poplar-type propolis compared with the common Chinese propolis, containing more benzyl *p*-coumarate and *p*-coumaric acid [26]. CBMP has a high commercial value. However, its bioactivity has not been explored.

The aim of this study was to investigate the antiproliferative effect of CBMP on cancer cells and its molecular mechanisms in cell signaling. Our results demonstrated that CBMP could suppress the growth of SGC-7901 cells by affecting important cell signaling pathways related to apoptosis and cell cycle.

## 2. Materials and Methods

### 2.1. Reagents

We obtained Dulbecco's Modified Eagle Medium (DMEM) and 0.25% Trypsin from Thermo Scientific HyClone (Logan, USA). Fetal bovine serum (FBS) was purchased from Gibco (New York, USA). Dimethyl sulfoxide (DMSO) was purchased from Sangon Biotechnology Co., Ltd. (Shanghai, China). Cell counting kit-8 (CCK-8) and Apoptosis Detection Kit were obtained from the Dojindo (Kumamoto, Japan). Cell Cycle Detection Kit was purchased from KeyGEN Biotech (Nanjing, China). We purchased anti-rabbit secondary antibodies and the primary antibodies against tubulin, cytochrome C (cytoplasm), B-cell lymphoma (Bcl-2), cleaved caspase 3, P53, Bid, Bax, cleaved caspase 9, cyclin-dependent kinase 2 (CDK2), cell division control protein 2 (CDC2), cleaved caspase 8, cleaved PARP, and cyclin B1 from Abcam (Cambridge, UK). We purchased primary antibodies against phosphorylated retinoblastoma protein (P-Rb), cyclin A2, cyclin E, and E2F1 from HUABIO Biotechnology (Hangzhou, China).

Caffeic acid, *p*-coumaric acid, CAPE, ferulic acid, isoferulic acid, kaempferol, 3,4-dimethoxycinnamic acid, pinocembrin, naringenin, quercetin, apigenin, chrysin, and galangin were obtained from Sigma-Aldrich (Missouri, USA). Pinobanksin, 3-O-acetyl pinobanksin, and benzyl caffeate were obtained from Haishu Apexocean Biochemicals (Ningbo, China), and benzyl *p*-coumarate was purchased from Kunming BioBioPha Co., Ltd. (Kunming, China).

### 2.2. Preparation of CBMP Extract

Propolis sample (voucher specimen No. CBM65) used in this study was collected by beekeepers in the Changbai Mountain area. The raw propolis was extracted at 45 min with 95% ethanol solution in an ultrasonic water bath; after that, the mixture was filtered and the residue was reextracted twice. Then, the filtrates were put in a refrigerator overnight to remove the insoluble matter and then evaporated to dryness. The dry propolis extract was redissolved in ethanol to obtain solutions at 50 and 10 mg/ml. The final concentration of ethanol in the cell culture was less than 0.5% (v/v). The solutions were purified with 0.22 *μ*m filters and, then, put in a refrigerator until use.

### 2.3. Chemical Analysis

The chemical composition of CBMP used in this study was analyzed by the high-performance liquid chromatography (HPLC) method reported earlier [26] using Agilent HPLC 1200 Series (Calif., USA) equipped with an HP-C18 column (5 *μ*m, 150 × 4.6 mm, Sepax Technologies, Del., USA) and column temperature at 33°C. CBMP sample (5 *μ*L) was injected by an automatic sampler system and monitored by a UV detector at the wavelength of 280 nm. Aqueous phase A (1% acetic acid) and organic phase B (anhydrous methanol) made up the eluent, and flow rate was maintained at 1 mL/min. The eluent was adjusted as follows: 15% to 35% (B) from 0 to 30 min, 35% to 44% (B) from 30 to 46 min, 44% to 50% (B) from 46 to 70 min, 50% to 52% (B) from 70 to 77 min, 52% to 60% (B) from 77 to 92 min, 60% to 75% (B) from 92 to 115 min, 75% to 100% (B) from 115 to 125 min, and 100% to 15% (B) from 125 to 135 min.

The contents of the identified compounds in CBMP were quantified using the regression equation of standard substances.

### 2.4. Cell Culture

The following cancer cell lines were obtained from the Cell Bank of Type Culture Collection of Chinese Academy of Sciences (Shanghai, China), including human pancreatic cancer cell PANC1, human lung cancer cell A549, human gastric cancer cell SGC-7901, human colon cancer cell HCT116, human liver cancer cell HepG2, human bladder cancer cell T24, and human breast cancer cell MDA-MB-231. Human embryonic kidney cell line HEK293 was a gift from Zhejiang Chinese Medical University and authenticated by STR analysis. All cells were cultured in DMEM supplemented with 10% (v/v) fetal bovine serum (FBS) in an incubator with standard cell culture conditions (37°C, 5% CO_2_).

### 2.5. Cell Viability Assay

We used the CCK-8 kit to measure cell viability according to the manufacturer's instructions. Cells (1 × 10^5^ cells/well) were seeded onto 96-well cell culture plates and cultured for 24 h before being treated with varying concentrations of CBMP for 24 h (from 6.25 *μ*g/mL to 75 *μ*g/mL), and the treated cells were incubated at 37°C for 2 h with 10 *μ*L of CCK-8. A microplate reader (Hercules, CA, USA) was used to measure absorbance at 450 nm.

### 2.6. Morphological Observation

SGC-7901 cells (1.5 × 10^5^ cells/well) were cultured in 6-well cell culture plates for 24 h. After they are treated with different concentrations of CBMP, the changes in cell morphology were observed by an Olympus phase-contrast microscope under 20x magnification.

### 2.7. Cell Apoptosis Assay

SGC-7901 cells (1.5 × 10^5^ cells/well) were cultured in 6-well cell culture plates for 24 h and then treated with different concentrations of CBMP for 24 h. After that, cells were harvested and treated with Annexin V-FITC/PI in the light of the Apoptosis Detection Kit protocol. The treated cells resuspended in 300 *μ*L of binding buffer. 5 *μ*L of Annexin V-FITC was added and incubated in the dark for 10 min. Then, 5 *μ*L of PI was added and incubated in the dark for 15 min. After that, for each sample, about 10000 treated cells were analyzed by the BD FACSVerse flow cytometer (Becton Dickinson, NJ, USA) at 488 nm excitation wavelength and 525/595 nm emission wavelength.

### 2.8. Cell Cycle Assay

SGC-7901 cells (1.5 × 10^5^ cells/well) were cultured in 6-well cell culture plates for 24 h and then treated with different concentrations of CBMP for 24 h. The cells were collected and fixed with 70% ethanol and then put in a refrigerator overnight at 4°C. After that, the cells were washed twice and treated with Propidium Iodide (PI) according to the Cell Cycle Analysis kit and incubated in the dark for 30 min. The treated cells (about 1 × 10^4^) were analyzed by the BD FACSVerse flow cytometer at 488 nm excitation wavelength and 595 nm emission wavelength.

### 2.9. Reactive Oxygen Species Detection (ROS)

We determined the ROS generation in SGC-7901 cells by ROS Assay Kit. Cells (1.5 × 10^5^ cells/well) were cultured in 6-well cell culture plates for 24 h and then treated with different concentrations of CBMP. After 24 h incubation, DCFH-DA (10 *μ*mol/L) was added and incubated for 30 min at 37°C. For each sample, about 10000 treated cells were washed thrice and determined by the BD FACSVerse flow cytometer at 488 nm excitation wavelength and 525 nm emission wavelength.

### 2.10. Measurement of Mitochondrial Membrane Permeability (MMP)

We used the JC-1 probe to detect the MMP changes in SGC-7901 cells after CBMP treatment. Cells (1.5 × 10^5^ cells/well) were cultured in 6-well cell culture plates for 24 h and then treated with different concentrations of CBMP for 24 h. Then, JC-1 (20 *μ*mol/L) was added to every well and incubated for 30 min at 37°C. For each sample, about 10000 treated cells were washed thrice and observed by the BD FACSVerse flow cytometer at 488 nm excitation wavelength and 530/585 nm emission wavelength.

### 2.11. Western Blotting Assay

SGC-7901 cells (1.5 × 10^5^ cells/well) in the logarithmic growth phase were cultured in 6-well cell culture plates for 24 h and then treated with different concentrations of CBMP for 24 h. We used RIPA mixed with protease and phosphatase inhibitors to lyse to gain total proteins of SGC-7901 cells. The BCA protein assay kit (Fudebio, Hangzhou, China) was used to measure protein concentration. The extracted protein was packed and stored at −80°C. We used 10% or 12% SDS-PAGE to separate proteins and then transfer them onto polyvinylidene difluoride (PVDF) membranes (Millipore, Atlanta, USA). 5% skim milk was used to block PVDF membranes at room temperature for 1 h, and then, primary antibodies were incubated overnight in a refrigerator at 4°C. After that, the PVDF membranes were incubated with secondary antibodies for 2 h at room temperature. The signals were detected by ImmobilonWestern Chemiluminescent HRP Substrate (Millipore, Atlanta, USA), and the images of the band were evaluated using ImageJ software.

### 2.12. Statistical Analysis

We used GraphPad Prism 6 (San Diego, USA) to conduct statistical analyses. Each assay was repeated at least three times. The mean and standard deviation (SD) were calculated using the Microsoft Excel 2016 software (Redmond, USA). Data were shown as mean ± SD. We used Student's *t*-test to compare the control with each treatment (^*∗*^*P* < 0.05; ^*∗∗*^*P* < 0.01; ^*∗∗∗*^*P* < 0.001).

## 3. Results

### 3.1. Chemical Analysis of CBMP

**Figure 1:**
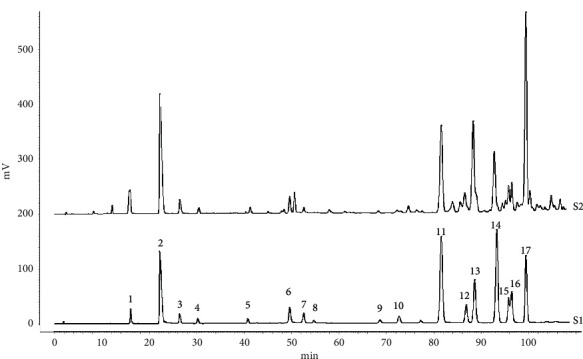
HPLC chromatograms of the standard solution (S1) and CMBP (S2): 1: caffeic acid; 2: p-coumaric acid; 3: ferulic acid; 4: isoferulic acid; 5: 3,4-dimethoxycinnamic acid; 6: pinobanksin; 7: naringenin; 8: quercetin; 9: kaempferol; 10: apigenin; 11: pinocembrin; 12: benzyl caffeate; 13: 3-O-acetyl pinobanksin; 14: chrysin; 15: CAPE; 16: galangin; 17: benzyl p-coumarate.

Sixteen phenolic compounds were identified by comparing their retention time and UV spectrum with standard phenolic compounds (Figure 1). The content of the main compounds in CBMP was quantified by the regression equation of standard substances (Table S1). In CBMP, abundant compounds are benzyl *p*-coumarate, *p*-coumaric acid, 3-O-acetyl pinobanksin, pinocembrin, and chrysin (Table 1).

### 3.2. Effects of CBMP on Cancer Cells Proliferation

**Figure 2:**
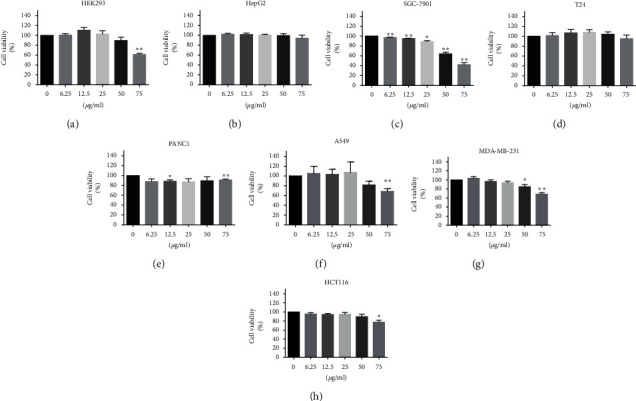
Cell proliferation evaluation by CCK-8 after different doses (from 6.25 *μ*g/mL to 75 *μ*g/mL) of CBMP treatment. (A–H) Various human cell lines. CBMP showed a weak activity against HepG2, T24, PANC1, A549, MDA-MB-231, and HCT116 cells but a strong activity against SGC-7901 cell. ^*∗*^*P* < 0.05; ^*∗∗*^*P* < 0.01 compared with the control group.

CBMP showed no inhibition effect on HEK293, HepG2, T24, and PANC1 cells and weak activities against A549, MDA-MB-231, and HCT116 cells at concentrations up to 50 *μ*g/mL (Figure 2). However, the cell viability of SGC-7901 cells was decreased significantly in a dose-dependent manner (Figure 2(c)). The IC_50_ value of CBMP against SGC-7901 cells was 66.64 *μ*g/mL.

**Figure 3:**
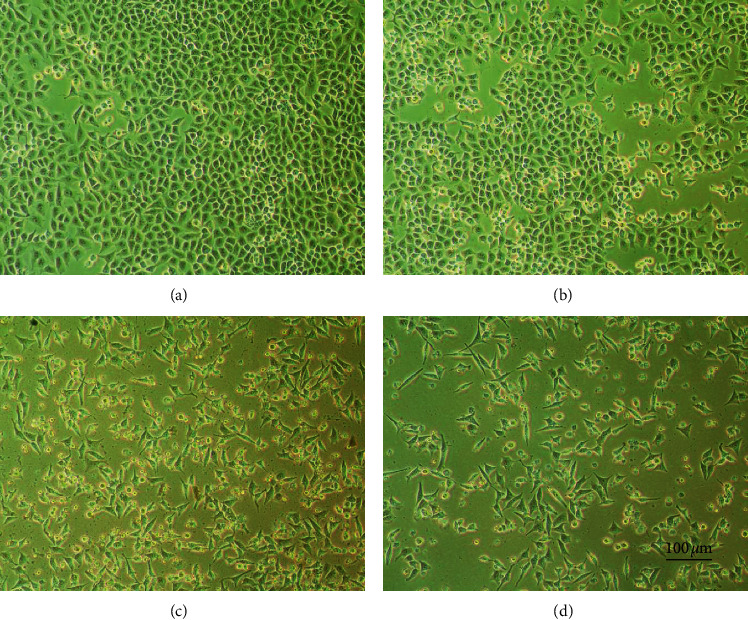
Changes in cell morphology after treatment with different concentrations of CBMP: (a) control; (b) 12.5 *μ*g/mL; (c) 25 *μ*g/mL; (d) 50 *μ*g/mL. Scale bar = 100 *μ*m.

The morphological changes of SGC-7901 cells after CBMP treatment were also observed. The normal SGC-7901 cells were flattened and grew closely attached (Figure 3(a)). CBMP treatment caused SGC-7901 cells to shrink, loose, and reduce in number and at high concentrations caused large numbers of cells to float (Figures 3(b)–3(d)). This result indicates that CBMP could inhibit cell proliferation and possibly induce cell apoptosis in SGC-7901.

### 3.3. CBMP Promoted ROS Generation and Loss of MMP

**Figure 4:**
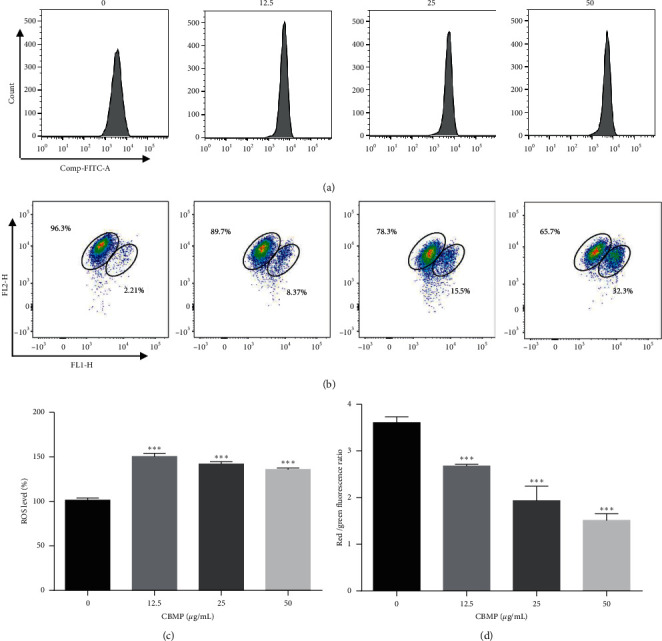
CBMP induced ROS generation and MMP loss in SGC-7901 cells. (a) SGC-7901 cells were treated with CBMP (12.5 *μ*g/mL, 25 *μ*g/mL, or 50 *μ*g/mL) for 24 h and stained by DCFH-DA and then measured by flow cytometry. (b) The changes of MMP in SGC-7901 after treatment with CBMP (12.5 *μ*g/mL, 25 *μ*g/mL, or 50 *μ*g/mL) for 24 h were measured by flow cytometry. (c) The changes in the ROS level in SGC-7901 cells after treatment with CBMP for 24 h were indicated by histograms. (d) Red/green fluorescence ratio was indicated by histograms, and the ratio was decreased significantly, indicating the loss of MMP in SGC-7901 cells. ^*∗∗∗*^*P* < 0.001 versus the control group.

Disproportional increase of ROS in cancer cells has been reported to affect many characters of cell behavior such as cell proliferation, cell morphology, cell cycle, and apoptosis [27, 28]. Compared with control cells, the production of ROS of SGC-7901 cells showed a significant increase in the treated group under different concentrations of CBMP (Figures 4(a) and 4(c)). This result indicates that CBMP induces ROS generation in SGC-7901, which may be an important way to exert biological anticancer activity.

ROS are produced mainly in mitochondria and their accumulation can lead to mitochondrial dysfunction such as the depolarization of MMP [29, 30]. After treatment with different concentrations of CBMP, the number of cells with normal MMP decreased (*P* < 0.001) in a dose-dependent manner (Figure 4(b)). Furthermore, the red/green fluorescence ratio was also decreased significantly, indicating the loss of MMP in SGC-7901 cells (Figure 4(d)).

### 3.4. CBMP Induces Apoptosis in SGC-7901 Cells

**Figure 5:**
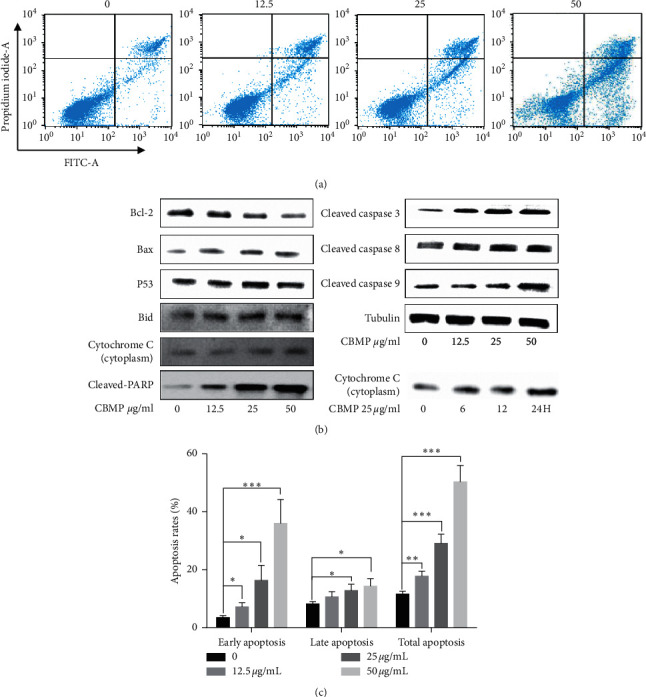
CBMP induced apoptosis in CBMP-treated SGC-7901 cells. (a) Cells were treated with CBMP (12.5 *μ*g/mL, 25 *μ*g/mL, or 50 *μ*g/mL) for 24 h and analyzed by flow cytometric assay after staining with Annexin V-FITC/PI. (b) The expression of proteins associated with apoptosis in CBMP-treated SGC-7901 cells was analyzed by Western blot. (c) Quantitative analysis of the cell different stages of apoptosis distributions; after 24 h treatment of CBMP, the early apoptosis rate was increased from 7.15 ± 1.22% to 36.05 ± 6.7% and late apoptosis rate increased from 10.64 ± 1.46% to 14.28 ± 2.11%. Data were expressed as mean ± SD (*n* = 3). ^*∗*^*P* < 0.05; ^*∗∗*^*P* < 0.01, ^*∗∗∗*^*P* < 0.001 versus the control group.

After 24 h treatment of CBMP, the cell apoptosis rates were increased from 17.79 ± 1.36% to 50.33 ± 4.59% (the early apoptosis rate was increased from 7.15 ± 1.22% to 36.05 ± 6.7% and late apoptosis rate increased from 10.64 ± 1.46% to 14.28 ± 2.11%), compared to 11.63 ± 0.78% in the control group (*P* < 0.001) (Figures 5(a) and 5(c)). To investigate the molecular mechanisms underlying this phenomenon, we used Western blot to analyze the key proteins associated with apoptosis in CBMP-exposed SGC-7901 cells. We found that the proapoptosis proteins Bax and Bid were upregulated, while the antiapoptosis protein Bcl-2 was downregulated (Figure 5(b)). The release of cytochrome C from mitochondria to the cytoplasm was observed, as well as the activation of cleaved caspase 8, cleaved caspase 9, cleaved caspase 3, and cleaved PARP (Figure 5(b)). Meanwhile, proapoptotic protein P53 was also activated after CBMP treatment.

### 3.5. CBMP Induces S Phase Arrest in SGC-7901 Cells

**Figure 6:**
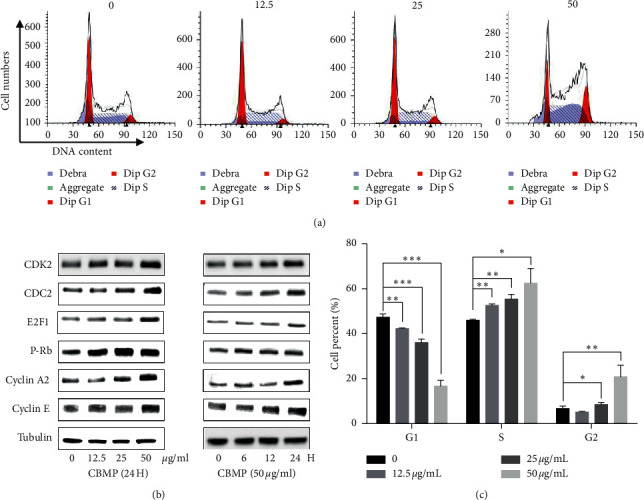
CBMP induced cell cycle arrest in S phase in SGC-7901 cells. (a) CBMP (12.5 *μ*g/mL, 25 *μ*g/mL, or 50 *μ*g/mL) treated cells were stained with PI and analyzed by flow cytometric. (b) The expression of key proteins in CBMP-treated cells associated with cell arrest in the S phase was analyzed by Western blot. (c) Quantitative analysis of cell numbers in different cell cycles of phase distributions; after 24 h treatment of CBMP, the proportion of S phase cells was increased from 46.04 ± 0.32% to 62.46 ± 5.39%, while G1 phase cells decreased from 47.28 ± 1.26% to 16.69 ± 2.12%. Data were expressed as mean ± SD (*n* = 3) ^*∗*^*P* < 0.05; ^*∗∗*^*P* < 0.01, ^*∗∗∗*^*P* < 0.001 versus the control group.

After CBMP treatment for 24 h, the proportion of S phase cells was remarkably (*P* < 0.001) increased from 46.04 ± 0.32% to 62.46 ± 5.39%, while G1 phase cells decreased from 47.28 ± 1.26% to 16.69 ± 2.12% (Figures 6(a) and 6(c)). The expression of factors proteins associated with the cell cycle was analyzed by Western blot. SGC-7901 cells treated with CBMP had a significant dose- and time-dependent upregulation of P-Rb, CDC2, CDK2, cyclin E, cyclin A, and E2F1 expressions (Figure 6(b)).

## 4. Discussion

Propolis is a widely used bee product with broad biological activities including antitumor properties [10]. CBMP is a unique poplar-type Chinese propolis [26]. In this study, we revealed that CBMP could specifically restrain the proliferation of SGC-7901 cells by promoting cell apoptosis and inducing S phase arrest. Different types of propolis have been shown to inhibit the proliferation of melanoma [31], liver cancer [32], and breast cancer [33]. To our knowledge, this is the first study on the antitumor property and molecular mechanism of actions of Chinese propolis on human gastric cancer cells.

Firstly, we examined the cytotoxicity of CBMP against seven different kinds of cancer cells. CBMP exerted unique activity against SGC-7901 cells at concentrations from 6.25 to 50 *μ*g/mL, without obvious inhibition of other cancer cell lines tested. The selectivity of CBMP on SGC-7901 may be related to its chemical compositions [34]. This is consistent with previous studies, as compounds isolated from Mexican propolis also showed selectivity when evaluated against six different cancer cell lines [35].

Subsequently, we studied the possible molecular mechanism of CBMP against SGC-7901 cell proliferation. Apoptosis is a key mechanism in which anticancer drugs kill cancer cells, which have mitochondria-mediated and death receptor-induced pathway [36]. Caspase family proteins play vital roles in apoptosis, and Bcl-2 family proteins can regulate the delicate caspase-cascade system [37, 38]. The finding of the present study showed that CBMP induced apoptosis of SGC-7901 cells indicating that the proapoptosis effect of CBMP is involved in the inhibition of SGC-7901 proliferation. This is further supported by the findings that CBMP strongly increased the expression of apoptosis proteins in SGC-7901 cells, including cleaved caspase 8, cleaved caspase 3, cleaved caspase 9, and cleaved PARP expressions, indicating the involvement of the death receptor-induced apoptosis pathway in SGC-7901 cells [39, 40]. Propolis also could induce apoptosis through upregulation of death receptor expression in prostate cancer cells [41, 42] and cervical cancer cells [43]. Moreover, increased expression levels of cytochrome C content in the cytoplasm (Figure 5(b)) are linked to an increase in mitochondrial oxidative stress [44]. Meanwhile, the expressions of the proapoptotic protein Bax and Bid were remarkably upregulated, whereas the antiapoptotic protein Bcl-2 was downregulated (Figure 5(b)). These results suggest that mitochondria-mediated pathway apoptosis was activated in SGC-7901 cells after the treatment with CBMP. The evidence of CBMP's action on mitochondria is further supported by the findings that CBMP caused excessive production of ROS and depolarization of MMP. ROS generation has been related to mitochondrial apoptosis and mitochondrial dysfunctions [29, 45]. Chinese propolis ethanolic extract has been reported to induce apoptosis in MCF-7 and MDA-MB-231 cells by ROS-dependent mitochondrial pathway [46].

Cell growth is regulated by cell cycle regulators [47]. In the present study, CBMP induced cell cycle arrest in the S phase (Figure 6), indicating that it affected cell cycle regulators. G1–S transcription is regulated by the expression of P-Rb, E2F, cyclins, CDK2, and CDC2 [48, 49]. CBMP induced Rb underwent phosphorylation; subsequently, P-Rb activated the expression of E2F1. CDK2, cyclin A, and cyclin E could form complexes and are activated by E2F1. The complexes cyclin E/CDK2 and cyclin A/CDK2 could regulate cell cycle by control G1 to S phase entry [50, 51]. CDC2 can also bind to cyclin E or cyclin B and promote the G1/S transition in parallel [52]. Thus, it is likely that the key kinase complex cyclin E/CDK2, cyclin A/CDK2, cyclin E/CDC2, and cyclin A/CDC2 are involved in the S phase arrest induced by CBMP. In addition, previous studies have demonstrated that ROS can upregulate cyclin levels and accelerate G1 to S phase transition [53]. Hence, CBMP causes excessive production of ROS in SGC-7901, which may be related to the cell cycle in the S phase induced by CBMP.

## 5. Conclusions

**Figure 7:**
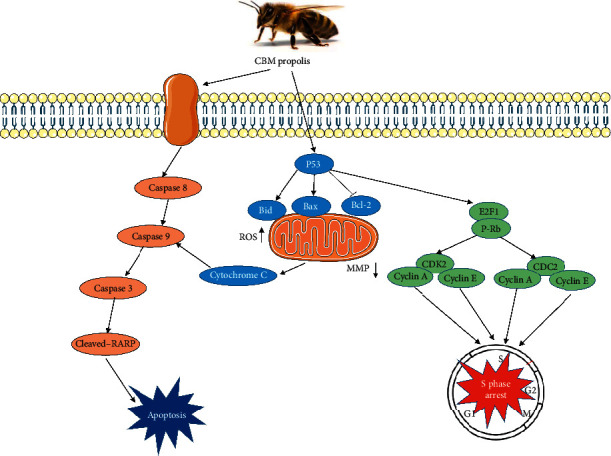
The signaling effects of CBMP on SGC-7901 cells.

Taken together, this study demonstrates that Chinese propolis from the Changbai Mountains selectively inhibits the proliferation of human gastric cancer SGC-7901 cells and induces apoptosis by activating death receptor-induced and mitochondria-mediated apoptosis pathways and blocks the cell cycle in S phase (Figure 7). Therefore, CBMP as a natural product has the potential to be used as a complementary medicine for cancer management. Further research is warranted to understand the role of the major component, benzyl *p*-coumarate, in the anticancer activities of CBMP.

## Figures and Tables

**Table 1:** The content of major compounds in CBMP.

Peak no.	Compound	Content (mg/g, mean ± SD)
1	Caffeic acid	3.31 ± 0.08
2	*p*-Coumaric acid	35.24 ± 0.51
3	Ferulic acid	4.46 ± 0.08
4	Isoferulic acid	1.64 ± 0.01
5	3,4-Dimethoxycinnamic acid	1.35 ± 0.02
6	Pinobanksin	5.94 ± 0.03
7	Naringenin	1.16 ± 0.02
8	Quercetin	—
9	Kaempferol	1.15 ± 0.01
10	Apigenin	0.77 ± 0.01
11	Pinocembrin	34.88 ± 0.96
12	Benzyl caffeate	15.20 ± 0.45
13	3-O-Acetyl pinobanksin	28.89 ± 0.13
14	Chrysin	16.32 ± 0.16
15	CAPE	11.36 ± 0.04
16	Galangin	11.92 ± 0.40
17	Benzyl *p*-coumarate	69.02 ± 0.52

## Data Availability

All data used to support the findings of this study are included within the article, and further details are available from the corresponding author.

## Supplementary Materials

Supplementary Materials Table S1: the regression equation of standard substances in CBMP.
