# Supplementary material for: Chinese Propolis Inhibits the Proliferation of Human Gastric Cancer Cells by Inducing Apoptosis and Cell Cycle Arrest
Source: Evid Based Complement Alternat Med. 2020 Jul 22;2020:2743058. doi: 10.1155/2020/2743058 (PMC7396018; doi:10.1155/2020/2743058)
Supplement: Supplementary Materials — Table S1: the regression equation of standard substances in CBMP. [file 2743058.f1.docx]

**Table S1.** The regression equation of standard substances in CBMP.

| **Compound** | **Regression equation** | **R²** |
| --- | --- | --- |
| Caffeic acid | y = 19.961x - 4.644 | R² = 0.9974 |
| *p*-Coumaric acid | y = 18.821x - 9.5091 | R² = 0.9976 |
| Ferulic acid | y = 14.423x - 0.2291 | R² = 0.9975 |
| Isoferulic acid | y = 14.930x + 3.201 | R² = 0.9969 |
| 3,4-Dimethoxycinnamic acid | y = 14.644x + 4.468 | R² = 0.9967 |
| Pinobanksin | y = 15.701x - 2.196 | R² = 0.9963 |
| Naringenin | y = 14.090x + 172.892 | R² = 0.9585 |
| Quercetin | y = 6.728x - 3.130 | R² = 0.9813 |
| Kaempferol | y = 10.468x - 20.948 | R² = 0.9846 |
| Apigenin | y = 13.507x - 14.146 | R² = 0.9929 |
| Pinocembrin | y = 17.531x + 25.354 | R² = 0.9970 |
| Benzyl caffeate | y = 9.729x + 0.049 | R² = 0.9972 |
| 3-Oacetyl pinobanksin | y = 23.389x + 14.159 | R² = 0.9969 |
| Chrysin | y = 24.474x - 28.395 | R² = 0.9977 |
| CAPE | y = 11.024x + 5.709 | R² = 0.9971 |
| Galangin | y = 10.560x - 35.330 | R² = 0.9980 |
| Benzyl *p*-coumarate | y = 14.182x + 11.489 | R² = 0.9999 |
